# Vascular Access Complexity and Thrombectomy Efficiency in Proximal MCA M1 Occlusion: Evaluation of the Bovine Aortic Arch–Dolichoarteriopathy Score

**DOI:** 10.3390/medicina62101933

**Published:** 2026-10-07

**Authors:** Mehtap Erdogan, Bilgehan Atılgan Acar, Mehmet Yıldız, Halil Alper Eryılmaz, Enes Zafer, Sena Boncuk Ulaş

**Affiliations:** 1 Department of Anatomy, Faculty of Medicine, Sakarya University, 54100 Sakarya, Türkiye; mehtaperdogan@sakarya.edu.tr; 2 Department of Neurology, Faculty of Medicine, Sakarya University, 54100 Sakarya, Türkiye; 3 Giresun Provincial Health Directorate, Bulancak Şehit Er Enver Erdogan Family Health Center, 28300 Giresun, Türkiye; drmehmetyildiz54@gmail.com; 4 Department of Neurology, Sakarya Training and Research Hospital, 54100 Sakarya, Türkiye; dr.alpereryilmaz@gmail.com; 5 Department of Internal Medicine, Sakarya Training and Research Hospital, 54100 Sakarya, Türkiye; ezafer@sakarya.edu.tr; 6 Lille Neuroendocrinology Team, Department of Lille Neuroscience and Cognition, University of Lille, 59000 Lille, France; sena.boncuk-ulas.etu@univ-lille.fr

**Keywords:** acute ischemic stroke, mechanical thrombectomy, B.A.D. score, middle cerebral artery, recanalization, puncture

## Abstract

*Background and Objectives:* In this study, it was aimed to investigate the relationship between the Bovine Aortic Arch–Dolichoarteriopathy (B.A.D.) score and the procedural characteristics of mechanical thrombectomy, recanalization success, and 90-day functional outcomes in patients with acute ischemic stroke with proximal M1 occlusion of the middle cerebral artery (MCA). *Materials and Methods:* A total of 220 adult patients who underwent mechanical thrombectomy for proximal MCA M1 occlusion were included in this retrospective, single-center cohort study. The patients were divided into good B.A.D. (<2; *n* = 143) and poor B.A.D. (≥2; *n* = 77) groups. Admission National Institutes of Health Stroke Scale (NIHSS) and Alberta Stroke Program Early CT Score (ASPECTS) scores, collateral status, thrombectomy time intervals, number of passes, first-pass effect (FPE), and Modified Thrombolysis in Cerebral Infarction (mTICI) recanalization grades were evaluated. The primary outcome was functional independence at day 90 (mRS 0–2). Associations with procedural and clinical outcomes were evaluated using multivariable logistic and linear regression analyses. *Results:* The puncture-to-recanalization time was significantly longer in the poor B.A.D. group [67 (50.5–88.0) min vs. 43 (32.0–65.0) min, *p* < 0.001], and the FPE rate was lower (37.7% vs. 51.7%, *p* = 0.046). After adjustment for thrombectomy technique and clinical covariates, poor B.A.D. remained independently associated with lower odds of FPE success (aOR = 0.439, 95% CI: 0.229–0.839; *p* = 0.013). In the procedurally adjusted linear regression model, poor B.A.D. also remained independently associated with longer puncture-to-recanalization time after additional adjustment for the number of thrombectomy passes and thrombectomy technique (B = 0.294, 95% CI: 0.188–0.400; *p* < 0.001). Final mTICI ≥ 2b, ≥2c, and 3 recanalization rates did not differ between the groups. Although 90-day functional independence was lower in the poor B.A.D. group, the difference was not significant (51.9% vs. 63.6%, *p* = 0.092), and no statistically significant independent association between B.A.D. status and 90-day functional independence was observed in the multivariable analysis. *Conclusions:* Poor B.A.D. anatomy was associated with greater procedural complexity during mechanical thrombectomy for proximal MCA M1 occlusion, including longer puncture-to-recanalization time and lower odds of FPE success, even after adjustment for available procedural and clinical factors. However, no statistically significant independent association was demonstrated between B.A.D. status and 90-day functional independence, and final recanalization rates did not differ significantly between the groups. These findings suggest that the B.A.D. score may have potential value as a preprocedural marker of vascular access complexity; however, prospective external validation is required before its use for procedural planning can be recommended.

## 1. Introduction

Acute ischemic stroke (AIS) continues to be one of the leading causes of permanent neurological disability and mortality, and large vessel occlusions (LVO) in particular constitute an important clinical burden. In occlusions in critical regions of the anterior circulation, such as the proximal M1 segment of the middle cerebral artery (MCA), mechanical thrombectomy (MT) has become the principal recanalization treatment that improves functional outcomes in appropriate patients. In the current American Heart Association/American Stroke Association guideline, endovascular thrombectomy is strongly recommended in patients with proximal LVO of the internal carotid artery or of the M1 segment who have appropriate clinical and imaging characteristics [1]. However, successful endovascular treatment is not limited to the opening of the occluded vessel; the achievement of recanalization in as short a time as possible and with the minimum number of attempts has an important role in the determination of the clinical prognosis of the patient.

The critical importance of time in mechanical thrombectomy has been demonstrated particularly by the fact that the prolongation of the interval between puncture and recanalization is unfavorably associated with functional outcomes. Delay in the reperfusion time may contribute to the emergence of clinically insufficient outcomes despite successful vessel opening, by increasing the risk of irreversible damage to the ischemic tissue. Indeed, in a multicenter study recently performed by Memiş et al., it was shown that futile recanalization developed in an important proportion of the patients in whom complete recanalization was obtained by mechanical thrombectomy performed for MCA occlusion, and that advanced age, longer puncture-to-recanalization time, poor collateral circulation, and a high 24 h NIHSS score were independently associated with this condition [2]. These findings show that not only the final recanalization grade but also the time elapsed in order to reach recanalization and the procedural process should be taken into consideration from a clinical point of view.

One of the important factors that may affect the duration and the technical success of endovascular treatment is the extracranial vascular anatomy of the patient. In particular, the aortic arch type, the bovine arch variation, tortuosity of the arch and of the internal carotid artery, and dolichoarteriopathy may make the advancement of catheter and guidewire systems to the target vessel difficult. The B.A.D. score developed by Snelling et al. aimed to define vascular anatomical difficulty in anterior circulation thrombectomy quantitatively by evaluating the bovine arch, the aortic arch type, and internal carotid artery dolichoarteriopathy together. In that study, it was shown that a high B.A.D. score was independently associated with a longer puncture-to-recanalization time and a longer first-pass time [3]. Similarly, studies examining extracranial vascular characteristics have shown that aortic arch and carotid artery anatomy may be effective on procedure duration, access to the target vessel, and recanalization success [4,5]. The present study therefore evaluates a previously described anatomical scoring framework in patients with proximal MCA M1 occlusion, rather than developing a new prediction score or deriving outcome-based weights for its components.

The clinical importance of these anatomical characteristics suggests that procedural difficulty in mechanical thrombectomy should not be evaluated only as a technical problem. Difficult vascular access may prolong the catheterization time, may require a greater number of device passes, and may cause a delay in recanalization. Therefore, just as the relationship between anatomical complexity and clinical outcome may not be direct, this relationship may also emerge to a large extent through the procedural time and the manner of reaching recanalization. In the study performed by Eryılmaz et al. in patients with acute basilar artery occlusion, it was also shown that the success of endovascular treatment may be affected by numerous clinical and procedural factors such as etiology, thrombectomy technique, and lesion localization [6]. For this reason, a holistic approach that evaluates the vascular anatomy together with the other determinants of the treatment process is necessary.

Although the final modified Thrombolysis in Cerebral Infarction (mTICI) grade is an important outcome measure in the evaluation of the technical success of mechanical thrombectomy, the recanalization level obtained only at the end of the procedure may not reflect the procedure in its entirety. Complete recanalization (mTICI 3) after a single thrombectomy pass without rescue treatment (first-pass effect, FPE) has gained particular importance because recanalization is reached in a shorter time and a smaller number of device passes is required.

Viewed from this aspect, whether the anatomical difficulty created by a high B.A.D. score during mechanical thrombectomy affects the final recanalization success or primarily the efficiency of the process of reaching recanalization is an important research question. In particular, the evaluation of the effect of a high B.A.D. score on FPE and on the puncture-to-recanalization time together with the final mTICI success and 90-day functional independence may provide a better understanding of how anatomical complexity is reflected in clinical outcomes.

However, studies in which the B.A.D. score is evaluated together with the procedural time, FPE, final recanalization, and 90-day functional outcomes in patients who undergo mechanical thrombectomy for occlusion of the proximal MCA M1 segment are limited. In particular, whether a high B.A.D. score, instead of directly decreasing the final recanalization success, affects the procedural efficiency by making access to the target vessel and the process of reaching recanalization difficult, and whether this effect is independently reflected in clinical outcomes, have not yet been sufficiently clarified.

In this study, we aimed to evaluate the relationship of the B.A.D. score with procedural and clinical outcomes in patients with acute ischemic stroke who underwent mechanical thrombectomy for proximal MCA M1 occlusion. In particular, we investigated the effect of the B.A.D. score on the puncture-to-recanalization time, on the number of thrombectomy passes, and on FPE; and, in addition, its relationship with the final recanalization success and with 90-day functional independence. The main hypothesis of our study was that a high B.A.D. score is related to the efficiency of the process of reaching recanalization rather than to the final recanalization success.

## 2. Materials and Methods

### 2.1. Study Design and Patient Population

This study is a single-center, observational, cohort-type research in which the clinical, laboratory, and radiological data of adult patients who had undergone mechanical thrombectomy for acute ischemic stroke and who had proximal occlusion in the M1 segment of the middle cerebral artery (MCA) were evaluated retrospectively. The study was carried out at the Stroke Center of the Department of Neurology of Sakarya University Training and Research Hospital. The data of the patients who presented with acute ischemic stroke to the Neurology Clinic of Sakarya Training and Research Hospital between 1 May 2020 and 1 May 2026 and who underwent mechanical thrombectomy were screened.

Patients who were 18 years of age and older, who had a diagnosis of acute ischemic stroke, in whom proximal occlusion in the MCA M1 segment was detected on vascular imaging, and to whom mechanical thrombectomy was applied because of this occlusion, were included in the study. All eligible patients meeting the inclusion criteria during the study period were consecutively screened and included according to the predefined eligibility criteria. Patients who did not have the necessary medical records related to the procedure and to the clinical outcomes, or whose day-90 modified Rankin Score (mRS) data could not be accessed, were not included in the study. In addition, patients who had posterior circulation occlusion, who had an accompanying vascular pathology that could markedly affect the vascular access for mechanical thrombectomy, such as tandem occlusion or severe cervicocranial stenosis, and whose basic clinical or radiological variables were missing to a degree that could not be evaluated, were excluded.

**Figure 1:**
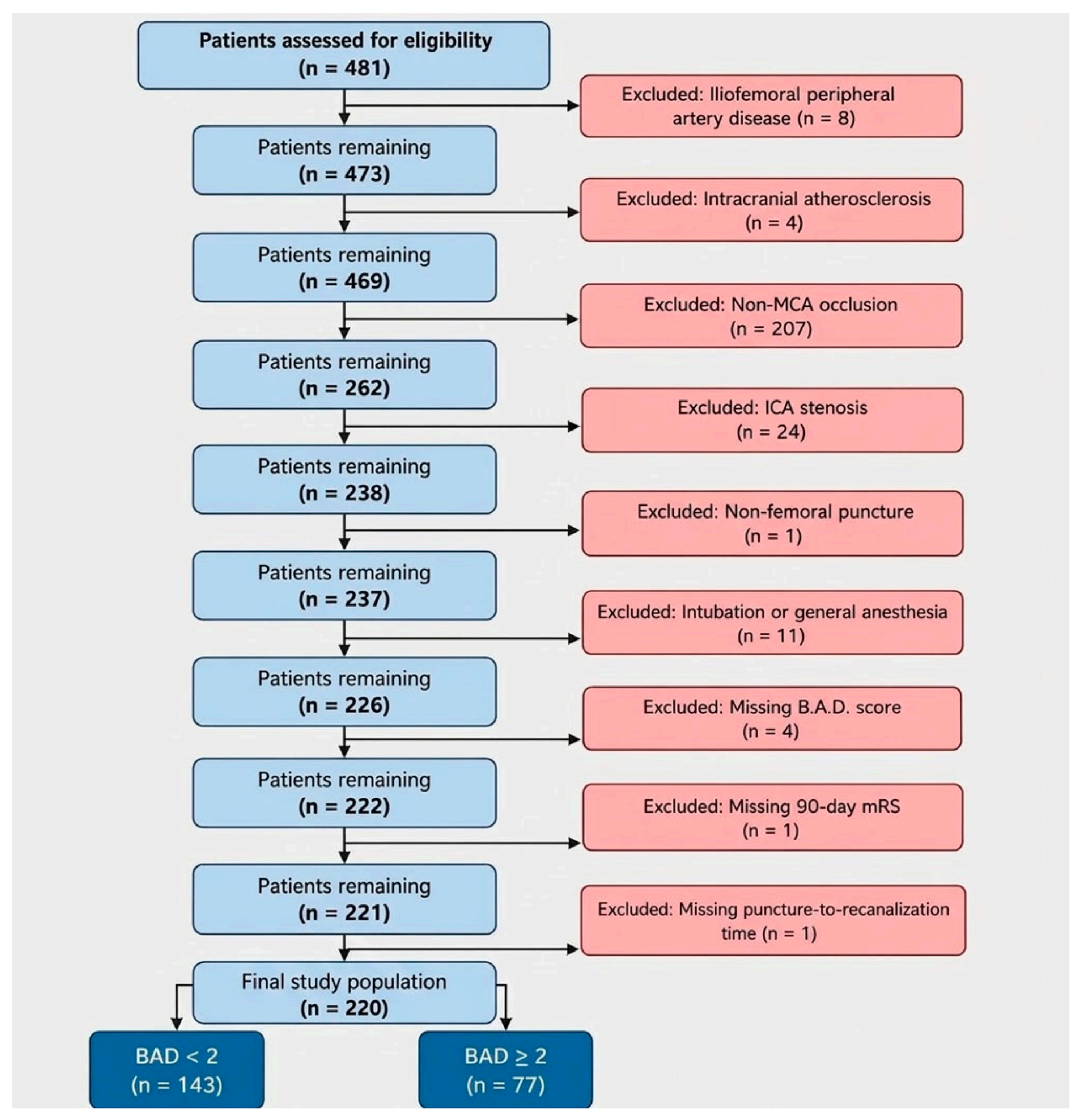
Flowchart of Study Population Selection.

A total of 481 patients were assessed for eligibility. As detailed in Figure 1, sequential exclusions comprised iliofemoral peripheral artery disease (*n* = 8), intracranial atherosclerosis (*n* = 4), non-MCA occlusion (*n* = 207), ICA stenosis (*n* = 24), non-femoral puncture (*n* = 1), intubation or general anesthesia (*n* = 11), missing B.A.D. score (*n* = 4), missing 90-day mRS data (*n* = 1), and missing puncture-to-recanalization time (*n* = 1).

Following these exclusions, 220 patients were included in the final analytical cohort: 143 in the good B.A.D. group (<2) and 77 in the poor B.A.D. group (≥2). Figure 1 presents the number of patients remaining after each screening stage.

### 2.2. Data Collection Method

The patient data were obtained through the hospital information management system (HIMS) and through PACS (Picture Archiving and Communication System), which is the radiological imaging archive. Within the scope of the research, no additional diagnostic or therapeutic intervention was applied to the patients.

### 2.3. Evaluation of Clinical and Laboratory Data

The age, sex, vascular risk factors, past medical history characteristics, and clinical data at the time of admission of the patients were recorded retrospectively through the HIMS. The stroke severity at the time of admission was evaluated using the National Institutes of Health Stroke Scale (NIHSS) [7]. Among the routine hemogram and biochemical parameters at admission, the available data such as hemoglobin, platelet count, glucose, urea, creatinine, sodium, potassium, low-density lipoprotein cholesterol (LDL-C), high-density lipoprotein cholesterol (HDL-C), C-reactive protein (CRP), estimated glomerular filtration rate (eGFR), and international normalized ratio (INR) were recorded.

Stroke etiologies were classified according to the Trial of Org 10172 in Acute Stroke Treatment (TOAST) classification as large artery atherosclerosis, cardioembolism, other determined etiology, and stroke of undetermined etiology [8].

Whether intravenous thrombolytic treatment had been applied from the onset of stroke until thrombectomy was also recorded.

### 2.4. Radiological Evaluation

The cranial computed tomography (CT) and CT angiography images of the patients, obtained before the procedure within the scope of the clinical routine, were evaluated retrospectively through PACS. On the imaging studies, the presence of proximal MCA M1 occlusion, the Alberta Stroke Program Early CT Score (ASPECTS) [9], the collateral circulation status, and the components of the B.A.D. score were evaluated.

The anatomical features used to calculate the B.A.D. score were assessed on preprocedural CT angiography images by a single interventional neurologist with 8 years of experience in interventional neurology. During B.A.D. scoring, the assessor was blinded to procedural and clinical outcomes, including puncture-to-recanalization time, FPE success, final angiographic recanalization grade, and 90-day modified Rankin Scale scores. All three anatomical components—bovine arch configuration, aortic arch type, and internal carotid artery dolichoarteriopathy—were evaluated by the same assessor. No independent second-reader assessment was performed; therefore, inter-rater agreement was not evaluated, and no disagreement-resolution procedure was applicable.

Collateral circulation was evaluated using the modified TAN collateral score (mTAN) [10], and mTAN 2–3 was accepted as good and mTAN 0–1 as poor collateral circulation.

### 2.5. Calculation of the B.A.D. Score

Vascular anatomical complexity was evaluated using the Bovine Aortic Arch–Dolichoarteriopathy (B.A.D.) score [3]. The score comprises three anatomical components: bovine arch configuration, aortic arch type, and internal carotid artery (ICA) dolichoarteriopathy. Each component was assessed on preprocedural CT angiography by the single blinded assessor described in Section 2.4.

**Figure 2:**
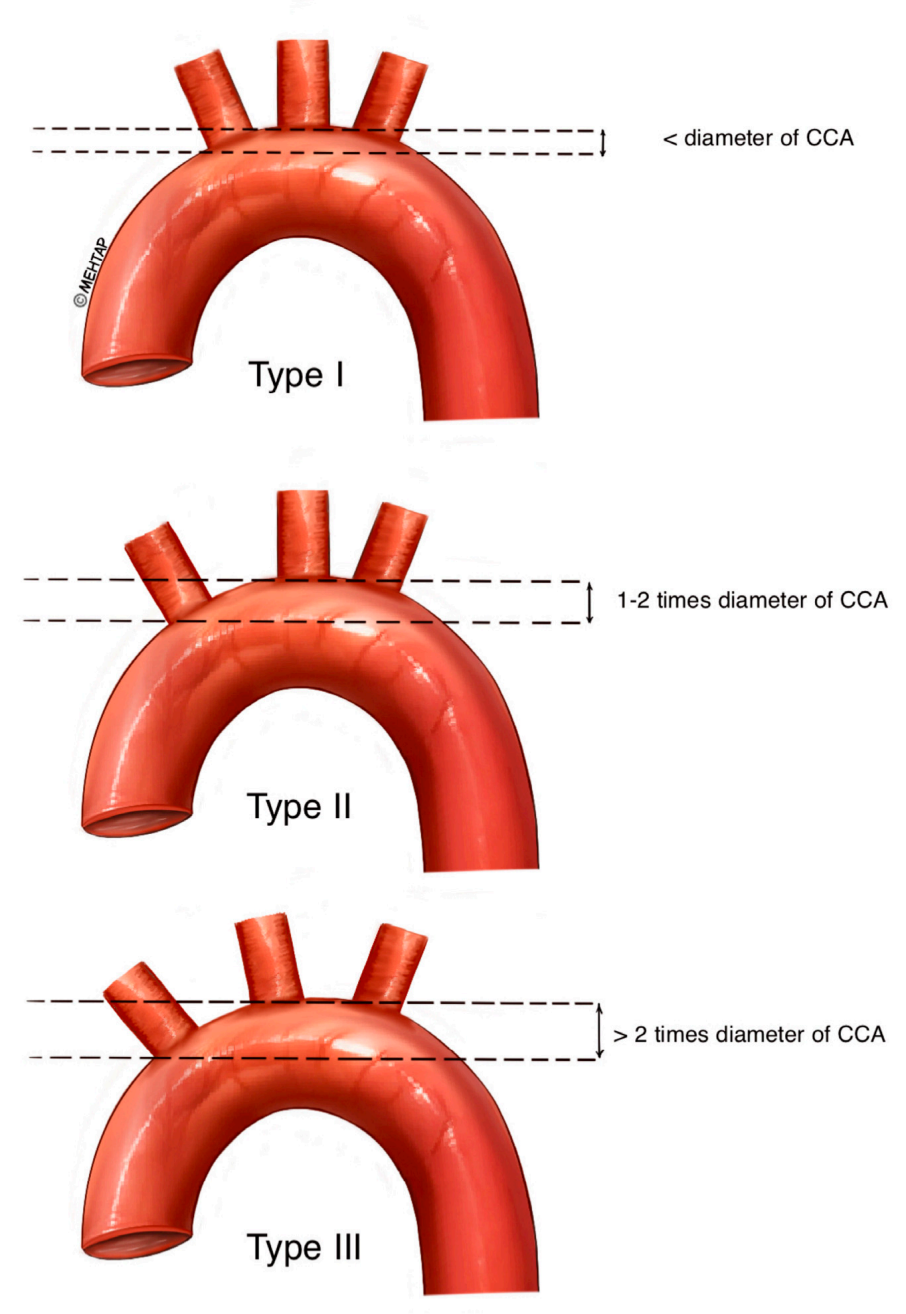
Components of the B.A.D. Score—Aortic Arch Morphology.

**Figure 3:**
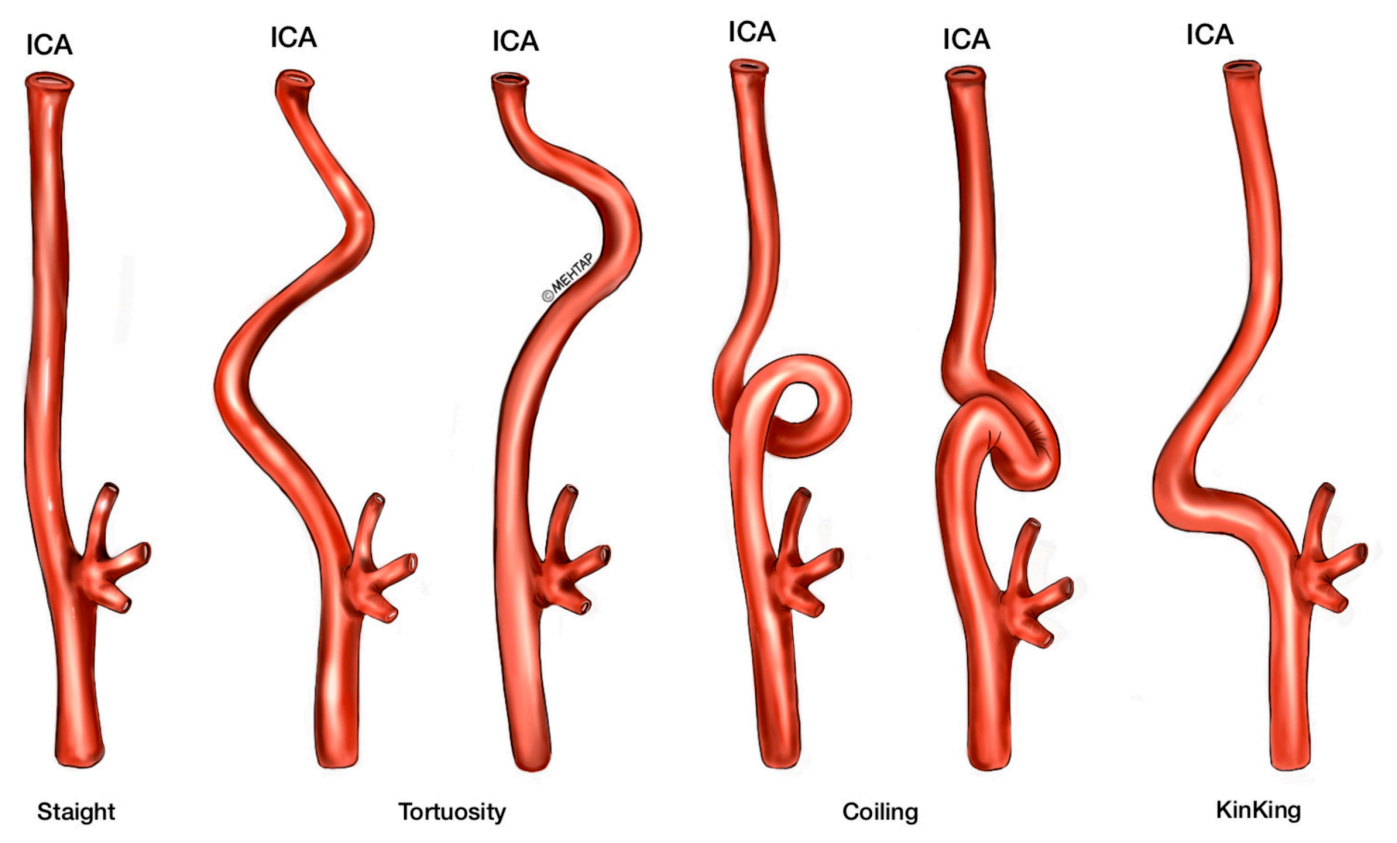
Components of the B.A.D. Score—ICA Variations.

**Figure 4:**
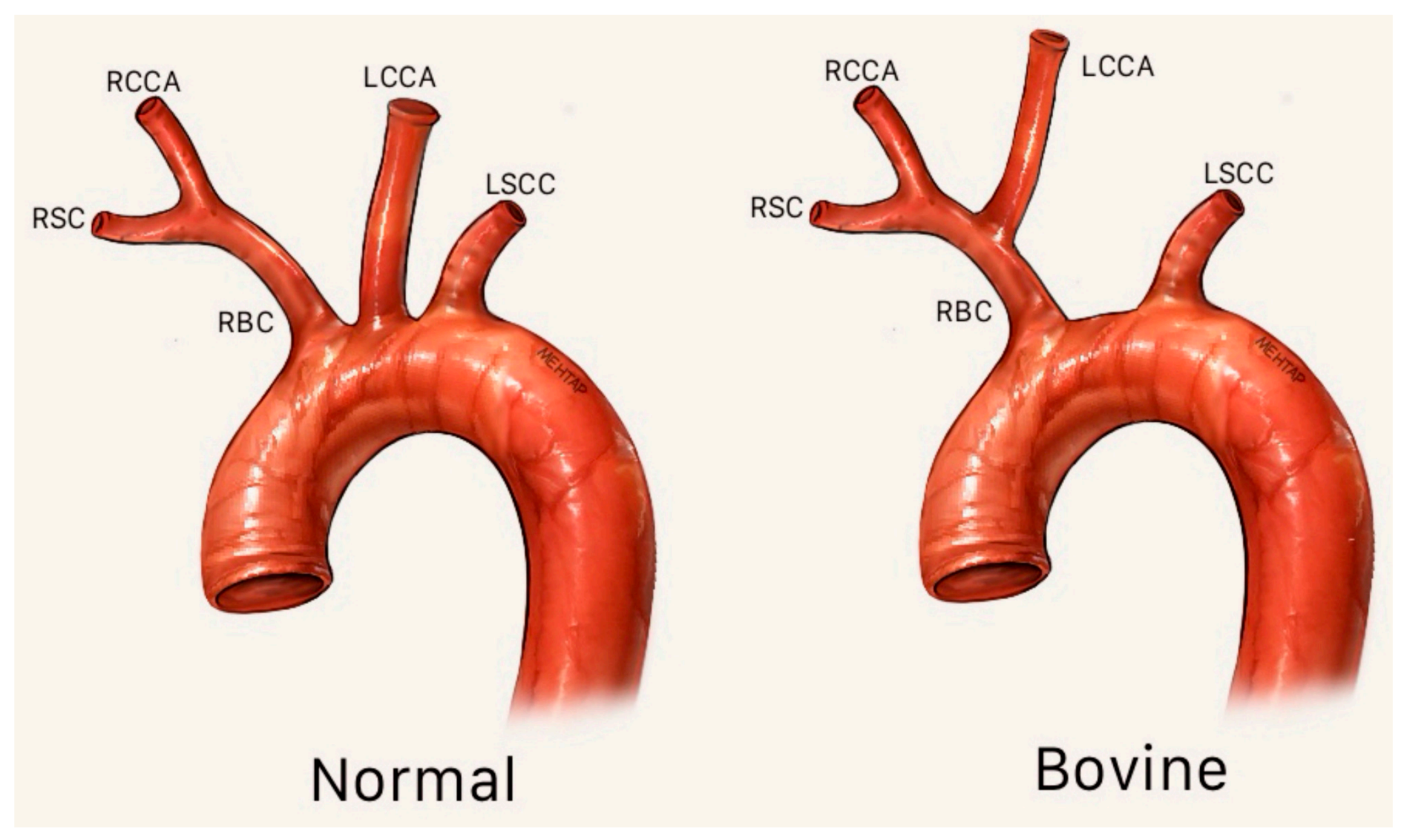
Components of the B.A.D. Score—Normal And Bovine Aortic Arch.

Bovine arch configuration was scored as 1 point when present and 0 points when absent. Aortic arch morphology was classified as Type I, Type II, or Type III and assigned 0, 1, or 2 points, respectively (Figure 2). ICA dolichoarteriopathy was scored as 0 points when absent or when only mild kinking was present, and as 1 point when moderate-to-severe kinking, tortuosity, or coiling was present (Figure 3). Normal and bovine arch configurations are illustrated in Figure 4. The component scores were summed to obtain a total B.A.D. score ranging from 0 to 4.

The maximum point contributions of the components differ: aortic arch type contributes up to 2 points, whereas bovine arch configuration and ICA dolichoarteriopathy each contribute up to 1 point. These point assignments are part of the previously described B.A.D. framework and were not derived from outcome-based weighting in the present cohort.

The B.A.D. score ranges from 0 to 4. For the analyses presented in this study, patients were classified as good B.A.D. (<2) or poor B.A.D. (≥2). This dichotomization was selected to maintain consistency with the previously described B.A.D. framework and to provide a clinically interpretable comparison of lower versus higher anatomical complexity. The cutoff was not derived from the present cohort and should not be interpreted as an independently validated clinical decision threshold.

### 2.6. Mechanical Thrombectomy and Procedural Variables

The angiographic and procedural variables related to mechanical thrombectomy were obtained by the retrospective analysis of medical records kept prospectively. The arterial puncture time, the final recanalization time, the puncture-to-recanalization time, the symptom-to-puncture time, and the symptom-to-recanalization time were evaluated. In addition, the number of thrombectomy passes, the FPE success, and the thrombectomy technique applied were evaluated. The recorded thrombectomy technique represented the technique used during the first thrombectomy pass and was classified as contact aspiration alone or a combined technique using contact aspiration together with a stent retriever. All thrombectomy procedures were performed by the same interventional neurologist with 8 years of experience in interventional neurology. No balloon-guide catheter was used, and all aspiration catheters had an inner lumen diameter of 6F.

The puncture-to-recanalization time was defined as the time between arterial puncture and successful final recanalization.

The final recanalization success was evaluated using the modified Thrombolysis in Cerebral Infarction (mTICI) classification [11]. In the analyses, the recanalization categories mTICI ≥ 2b, mTICI ≥ 2c, and mTICI 3 were used. In addition, mTICI 2c–3 was converted into a binary variable as optimal recanalization and was evaluated in the regression analyses. The FPE was defined as the achievement of complete recanalization at the mTICI 3 level with a single pass of the thrombectomy device, without the application of any rescue treatment [12].

### 2.7. Evaluation of Clinical Outcomes

The primary clinical outcome was determined as functional independence at day 90 (mRS 0–2). At day 90, mRS 0–2 was accepted as functional independence and mRS 3–6 as poor functional outcome.

Among the secondary clinical and safety outcomes were the 90-day mRS score, mRS 0–1, 90-day mortality, symptomatic intracranial hemorrhage, and other procedural complications. Other procedural complications were evaluated as vasospasm, vessel rupture, and dissection. In addition, an exploratory subgroup analysis was performed in the patients in whom mTICI ≥ 2c recanalization was obtained, with respect to the 90-day mRS score, mRS 0–1, mRS 0–2, and mortality.

### 2.8. Statistical Analysis

Statistical analyses were performed using IBM SPSS Statistics version 26.0 for Windows (IBM Corp., Armonk, NY, USA). The distributional characteristics of continuous variables were evaluated, and variables were presented, as appropriate, as mean ± standard deviation (SD) or median with interquartile range (IQR). Categorical variables were expressed as numbers and percentages. For comparisons between patients with Good B.A.D. (<2) and Poor B.A.D. (≥2), the independent-samples *t*-test was used for normally distributed continuous variables and the Mann–Whitney U test for non-normally distributed continuous variables. Categorical variables were compared using Pearson’s chi-square test or Fisher’s exact test, as appropriate.

For the primary group-based analyses, the B.A.D. score was dichotomized as <2 (Good B.A.D.) and ≥2 (Poor B.A.D.). B.A.D. status was entered as a binary variable in the primary regression models; therefore, the corresponding effect estimates represent comparisons between these two categories rather than the effect of a 1-point increase in the original B.A.D. score.

To identify factors associated with FPE success, univariate and multivariable binary logistic regression analyses were performed, with FPE success coded as 1 (yes) and 0 (no). The multivariable model included B.A.D. status (<2 vs. ≥2), age, sex, admission NIHSS, admission ASPECTS, collateral status (mTAN 0–1 vs. 2–3), and thrombectomy technique (aspiration alone vs. combined technique). Odds ratios (ORs) with 95% confidence intervals (CIs) were reported. Model calibration was assessed using the Hosmer–Lemeshow goodness-of-fit test, and overall model performance was additionally evaluated using the omnibus likelihood-ratio test and Nagelkerke R^2^.

Binary logistic regression analyses were also performed to evaluate factors associated with 90-day functional independence, defined as mRS 0–2. The dependent variable was coded as 1 for functional independence (mRS 0–2) and 0 for poor functional outcome (mRS 3–6). Model 1 included B.A.D. group, age, sex, admission NIHSS, admission ASPECTS, intravenous thrombolysis, and collateral status. Model 2 additionally included near-complete/complete recanalization (mTICI ≥ 2c), whereas Model 3 included puncture-to-recanalization time instead of mTICI ≥ 2c. ORs with 95% CIs were reported. Model fit was evaluated using the omnibus likelihood-ratio test, Hosmer–Lemeshow goodness-of-fit test, and Nagelkerke R^2^.

Because puncture-to-recanalization time was right-skewed, a natural logarithmic transformation was applied before linear regression analysis. Univariate linear regression analyses were first performed to evaluate the association of each predictor with log-transformed puncture-to-recanalization time. Two multivariable models were subsequently constructed. Model 1 included B.A.D. group (<2 vs. ≥2), age, sex, baseline NIHSS, baseline ASPECTS, intravenous thrombolysis, and collateral status. To further account for procedural characteristics that could influence procedural duration, Model 2 additionally included the number of thrombectomy passes and thrombectomy technique (aspiration alone vs. combined technique). Unstandardized regression coefficients (B) with 95% CIs were reported. Model fit was assessed using the F statistic, R^2^, and adjusted R^2^, and multicollinearity was evaluated using variance inflation factors (VIFs).

To assess the robustness of the findings to dichotomization of the B.A.D. score, sensitivity analyses were performed by replacing the binary B.A.D. classification (<2 vs. ≥2) with the original ordinal B.A.D. score (0–4), modeled per 1-point increase. The same covariate structures as in the corresponding primary multivariable models were retained: the FPE model included age, sex, admission NIHSS, admission ASPECTS, collateral status, and thrombectomy technique; the puncture-to-recanalization analyses retained both the clinically adjusted model (Model 1) and the procedurally adjusted model (Model 2); and the 90-day functional independence analyses retained the three models described above. These sensitivity analyses are presented in Supplementary Table S1.

Exploratory component-specific analyses were additionally performed to evaluate the individual associations of bovine arch, aortic arch type, and ICA dolichoarteriopathy with FPE, puncture-to-recanalization time, and 90-day functional independence. All three B.A.D. components were entered simultaneously into each corresponding multivariable model, with aortic arch Type I serving as the reference category. The component-specific models otherwise retained the covariate structures of the corresponding primary analyses, including procedural adjustment for thrombectomy technique in the first-pass model and for both thrombectomy technique and number of thrombectomy passes in the puncture-to-recanalization model. For 90-day functional independence, the component-specific model was adjusted for age, sex, admission NIHSS, admission ASPECTS, intravenous thrombolysis, and collateral status. These exploratory analyses are presented in Supplementary Table S2.

No imputation was performed for missing B.A.D. score, 90-day mRS, or puncture-to-recanalization time data; patients missing these key variables were excluded before formation of the final analytical cohort. For categorical predictors, where applicable, Good B.A.D. (<2), male sex, no intravenous thrombolysis, poor collateral status (mTAN 0–1), and aspiration alone were used as the reference categories. For continuous variables, ORs and regression coefficients represent the effect associated with a 1-unit increase in the corresponding variable. All statistical tests were two-sided, and *p* < 0.05 was considered statistically significant.

### 2.9. Ethical Approval

The study was conducted with the approval of the Scientific Research Ethics Committee of Health Sciences of Sakarya University. Because of the retrospective and non-interventional nature of the study, no additional diagnostic or therapeutic procedure was applied to the patients within the scope of the research. The clinical, imaging, and procedural data included in this study had been generated and prospectively recorded during routine clinical care before initiation of the present retrospective research. Ethical approval for the retrospective analysis was obtained before research-specific data extraction and statistical analysis were undertaken. No additional examination, intervention, or follow-up was performed for research purposes. The patient data were analyzed after being coded, and personal identity information was not included in the research data set. The study was conducted according to the guidelines of the Declaration of Helsinki and approved by the Scientific Research Ethics Committee of Health Sciences, Sakarya University Faculty of Medicine (Decision No: 712 and date of approval: 17 September 2026; Official Document No: E-43012747-050.04-627250).

## 3. Results

### 3.1. Study Population and B.A.D. Classification

To facilitate interpretation and future evidence synthesis, categorical outcomes are reported as absolute numbers and percentages for each B.A.D. group, while regression analyses are presented with effect estimates and 95% confidence intervals.

A total of 220 patients who underwent mechanical thrombectomy for proximal middle cerebral artery M1 occlusion were included in the study (Figure 1). According to the predefined Bovine Aortic Arch–Dolichoarteriopathy (B.A.D.) classification, 143 patients (65.0%) were in the good B.A.D. (<2) group and 77 patients (35.0%) in the poor B.A.D. (≥2) group.

The distribution of the anatomical components constituting the B.A.D. score is shown in Table 1. A bovine arch was detected in 31 patients (14.1%), all of whom were in the poor B.A.D. group. Aortic arch morphology was classified as Type I in 29 patients (13.2%), Type II in 141 patients (64.1%), and Type III in 50 patients (22.7%). Type I arch morphology was observed only in the good B.A.D. group, whereas Type III arch morphology was observed only in the poor B.A.D. group. Internal carotid artery dolichoarteriopathy was detected in 32 patients (14.5%), all of whom were in the poor B.A.D. group (Table 1).

The distribution of the original B.A.D. scores was as follows: score 0, 29 patients (13.2%); score 1, 114 (51.8%); score 2, 47 (21.4%); score 3, 24 (10.9%); and score 4, 6 (2.7%).

### 3.2. Demographic and Baseline Clinical Characteristics

The mean age of the study population was 68.79 ± 12.29 years. The patients in the poor B.A.D. group were significantly older than those in the good B.A.D. group (71.79 ± 10.51 vs. 67.17 ± 12.90 years; *p* = 0.007). No significant difference was found between the groups with respect to sex, vascular risk factors, history of previous stroke or transient ischemic attack, antithrombotic medication use, or blood pressure values at admission (Table 2).

Admission NIHSS and ASPECTS scores, the rate of intravenous thrombolysis, the affected hemisphere, and collateral circulation status were similar in the two B.A.D. groups (Table 3). The distribution of stroke etiologies also did not differ between the groups (*p* = 0.849). Cardioembolism was the most frequently detected etiology, in 142 patients (64.5%) (Table 4).

### 3.3. Procedural Characteristics and Reperfusion Outcomes

The procedural characteristics and angiographic outcomes are presented in Table 5. The median time from symptom onset to recanalization was 223.5 min (IQR, 182.8–311.8) in all patients and did not differ significantly between the B.A.D. groups [good B.A.D.: 221.0 min (IQR, 182.5–325.5); poor B.A.D.: 228.0 min (IQR, 186.0–309.0); *p* = 0.631].

In contrast, the time from puncture to recanalization was significantly longer in the poor B.A.D. group [67.0 min (IQR, 50.5–88.0) vs. 43.0 min (IQR, 32.0–65.0); *p* < 0.001]. The time from symptom onset to puncture did not differ significantly between the groups [good B.A.D.: 177.0 min (IQR, 126.0–254.0); poor B.A.D.: 160.0 min (IQR, 120.0–239.5); *p* = 0.313].

First-pass recanalization was achieved in 74 patients (51.7%) in the good B.A.D. group and in 29 patients (37.7%) in the poor B.A.D. group, and the difference between the groups was statistically significant (*p* = 0.046). The proportion of patients requiring three or more passes did not differ significantly between the groups (23.1% vs. 26.0%; *p* = 0.632). The median number of thrombectomy passes was 1 (IQR, 1–2) in the good B.A.D. group and 2 (IQR, 1–3) in the poor B.A.D. group, although this difference was not statistically significant (*p* = 0.075).

No significant difference was found between the B.A.D. groups with respect to final mTICI ≥ 2b, mTICI ≥ 2c, and mTICI 3 recanalization rates (*p* = 0.191, *p* = 0.194, and *p* = 0.114, respectively). The rate of use of the combined thrombectomy technique was also similar between the groups (61.5% vs. 58.4%; *p* = 0.654) (Table 5).

### 3.4. Clinical Outcomes and Procedural Complications

The day-90 clinical outcomes and procedural complications are presented in Table 6. The median 90-day mRS score was 2 (IQR, 0–5) in both B.A.D. groups, and no significant difference was found between the groups (*p* = 0.316).

Functional independence at day 90 (mRS 0–2) was achieved in 91 patients (63.6%) in the good B.A.D. group and in 40 patients (51.9%) in the poor B.A.D. group. Although the rate was numerically lower in the poor B.A.D. group, the difference did not reach statistical significance (*p* = 0.092). The rates of mRS 0–1 were also similar between the groups (49.7% vs. 42.9%; *p* = 0.336).

Ninety-day mortality was 20.3% in the good B.A.D. group and 20.8% in the poor B.A.D. group, and no significant difference was found between the groups (*p* = 0.930). The rates of symptomatic intracranial hemorrhage were 10.5% and 7.8%, respectively (*p* = 0.516). Other procedural complications were observed at a rate of 6.3% in the good B.A.D. group and 9.1% in the poor B.A.D. group (*p* = 0.446). These complications comprised vasospasm in 14 patients, vessel rupture in 1 patient, and dissection in 2 patients. Because vasospasm and dissection occurred concurrently in one patient, the total number of patients in whom a complication developed was calculated as 16.

### 3.5. Hematologic and Biochemical Parameters

The distribution of the hematologic and biochemical parameters according to B.A.D. group is presented in Table 7. No significant difference was found between the good and poor B.A.D. groups in any of the hemoglobin, platelet, leukocyte, neutrophil, lymphocyte, and monocyte values, or in the glucose, lipid profile, renal function test, CRP, and INR values (all *p* > 0.05).

### 3.6. Functional Outcomes in Patients Achieving Optimal Reperfusion

An exploratory subgroup analysis was performed in the 184 patients who achieved mTICI ≥ 2c recanalization. Of these patients, 123 were in the good B.A.D. group and 61 in the poor B.A.D. group (Table 8).

In this subgroup, the median 90-day mRS score was 1 (IQR, 0–3) in both B.A.D. groups, and no significant difference was found between the groups (*p* = 0.584). Functional independence (mRS 0–2) was achieved at a rate of 71.5% in the good B.A.D. group and 63.9% in the poor B.A.D. group (*p* = 0.293). The rates of excellent outcome at day 90 (mRS 0–1) were 56.9% and 54.1%, respectively (*p* = 0.718). Ninety-day mortality was found to be 16.3% in the good B.A.D. group and 18.0% in the poor B.A.D. group (*p* = 0.762).

### 3.7. Factors Associated with First-Pass Effect

In univariate analysis, Poor B.A.D. (≥2) was associated with lower odds of FPE compared with Good B.A.D. (<2) (OR = 0.563, 95% CI 0.320–0.992; *p* = 0.047). In the multivariable model adjusted for age, sex, admission NIHSS, admission ASPECTS, collateral status, and thrombectomy technique, Poor B.A.D. remained independently associated with lower odds of FPE (aOR = 0.439, 95% CI 0.229–0.839; *p* = 0.013). Female sex was independently associated with higher odds of FPE (aOR = 2.158, 95% CI 1.176–3.959; *p* = 0.013), whereas the combined technique, compared with aspiration alone, was independently associated with lower odds of FPE (aOR = 0.199, 95% CI 0.107–0.370; *p* < 0.001) (Table 9).

### 3.8. Predictors of Functional Independence at Day 90

The predictors of functional independence, defined as mRS 0–2 at day 90, were assessed by logistic regression analysis (Table 10). In the univariate analysis, although poor B.A.D. was associated with a lower likelihood of functional independence, this association did not reach the level of statistical significance (OR, 0.618; 95% CI, 0.352–1.084; *p* = 0.093).

After adjustment for the baseline variables in Model 1, the adjusted OR for poor B.A.D. was 0.578 (95% CI, 0.310–1.079; *p* = 0.085). In Model 2, in which final recanalization status was added to the model, the adjusted OR for poor B.A.D. was found to be 0.615 (95% CI, 0.309–1.226; *p* = 0.167). In Model 3, in which the procedural time variables were also taken into account, no significant association was found between poor B.A.D. and functional independence (adjusted OR, 0.896; 95% CI, 0.452–1.774; *p* = 0.752).

In Model 3, a higher admission NIHSS score was independently associated with a lower likelihood of 90-day functional independence (adjusted OR, 0.843; 95% CI, 0.768–0.926; *p* < 0.001). A longer time from puncture to recanalization was also found to be independently associated with a decrease in the likelihood of functional independence (adjusted OR, 0.977; 95% CI, 0.966–0.989; *p* < 0.001).

Model 1 [Omnibus χ^2^(7) = 33.911; *p* < 0.001; Hosmer–Lemeshow *p* = 0.226; Nagelkerke R^2^ = 0.195], Model 2 [Omnibus χ^2^(8) = 74.341; *p* < 0.001; Hosmer–Lemeshow *p* = 0.332; Nagelkerke R^2^ = 0.390], and Model 3 [Omnibus χ^2^(8) = 50.083; *p* < 0.001; Hosmer–Lemeshow *p* = 0.438] were statistically significant (Table 10).

### 3.9. Predictors of the Time from Puncture to Recanalization

Because puncture-to-recanalization time showed a right-skewed distribution, the variable was natural logarithm-transformed before linear regression analysis. In the clinically adjusted model (Model 1), Poor B.A.D. (≥2) was independently associated with longer puncture-to-recanalization time (B = 0.338, 95% CI 0.203–0.473; *p* < 0.001). After additional adjustment for the number of thrombectomy passes and thrombectomy technique (Model 2), the association between Poor B.A.D. and longer puncture-to-recanalization time remained statistically significant, although the coefficient was attenuated (B = 0.294, 95% CI 0.188–0.400; *p* < 0.001). In Model 2, a greater number of thrombectomy passes (B = 0.208, 95% CI 0.168–0.248; *p* < 0.001) and the combined technique compared with aspiration alone (B = 0.137, 95% CI 0.030–0.245; *p* = 0.013) were also independently associated with longer puncture-to-recanalization time. Model 2 was statistically significant [F(9,208) = 22.667, *p* < 0.001], explaining 49.5% of the variance in log-transformed puncture-to-recanalization time (R^2^ = 0.495; adjusted R^2^ = 0.473). No problematic multicollinearity was observed (VIF range, 1.058–1.171) (Table 11).

### 3.10. Sensitivity and Exploratory Component-Specific Analyses

Sensitivity analyses treating the B.A.D. score as an ordinal variable yielded findings generally consistent with the primary analyses (Supplementary Table S1). Each 1-point increase in the B.A.D. score was associated with longer puncture-to-recanalization time in both the clinically adjusted model (B = 0.181, 95% CI 0.110–0.251; *p* < 0.001) and the model additionally adjusted for the number of thrombectomy passes and thrombectomy technique (B = 0.164, 95% CI 0.110–0.218; *p* < 0.001). For FPE, the association was directionally consistent with the primary analysis but did not reach statistical significance (aOR = 0.721, 95% CI 0.514–1.010; *p* = 0.057). No statistically significant association was observed between the ordinal B.A.D. score and 90-day functional independence across the three adjusted models (all *p* > 0.05).

In exploratory component-specific analyses (Supplementary Table S2), Type III aortic arch and ICA dolichoarteriopathy were independently associated with longer puncture-to-recanalization time after adjustment for the other B.A.D. components and relevant clinical and procedural covariates (Type III vs. Type I: B = 0.409, 95% CI 0.207–0.612; *p* < 0.001; ICA dolichoarteriopathy: B = 0.199, 95% CI 0.047–0.351; *p* = 0.010). Bovine arch and Type II aortic arch were not significantly associated with puncture-to-recanalization time. None of the individual B.A.D. components showed a statistically significant independent association with FPE or 90-day functional independence.

## 4. Discussion

In this study, the relationship of the Bovine Aortic Arch–Dolichoarteriopathy (B.A.D.) score with procedural and clinical outcomes was evaluated in 220 patients who underwent mechanical thrombectomy for proximal MCA M1 occlusion. The main finding of our study is that a poor B.A.D. score is independently associated with a longer puncture-to-recanalization time and with a lower FPE success. In contrast, the final recanalization rates did not differ between the B.A.D. groups, and an independent relationship of the B.A.D. score with the 90-day functional outcome could not be demonstrated. The puncture-to-recanalization time was found to be 67 min in the poor B.A.D. group and 43 min in the good B.A.D. group, and this relationship also persisted in the multivariable analysis. Similarly, the FPE rate was 37.7% in the poor B.A.D. group and 51.7% in the good B.A.D. group, and poor B.A.D. was found to be independently associated with a lower first-pass success.

The 24 min difference in median puncture-to-recanalization time between the groups may be clinically relevant. In Model 3, longer puncture-to-recanalization time was associated with lower odds of 90-day functional independence (aOR per 1 min increase, 0.977; 95% CI, 0.966–0.989; Table 10). However, this association does not quantify the neurological impact attributable specifically to the between-group time difference. Neither symptom-to-recanalization time nor 90-day functional independence differed significantly between the B.A.D. groups, although these findings do not exclude clinically important differences. Given the observational design and the possibility of residual confounding despite adjustment for available procedural factors, the observed difference should be interpreted as an adjusted association rather than evidence that unfavorable anatomy caused worse neurological outcomes.

Independent studies have also demonstrated that unfavorable supra-aortic and carotid anatomy may increase access difficulty and prolong endovascular procedure times. Kaymaz et al. showed that carotid tortuosity significantly influenced internal carotid artery access time during mechanical thrombectomy, while Knox et al. demonstrated that aortic arch and great-vessel anatomy affected procedural performance in anterior circulation thrombectomy. More recently, Koge et al. reported that ICA tortuosity was associated with longer puncture-to-reperfusion time and a lower likelihood of FPE [13,14].

The principal study that enabled the development of the B.A.D. score was performed by Snelling et al. [3], and it was shown that a high score was associated with a longer recanalization time and with worse outcomes in anterior circulation thrombectomy. Supporting this mechanistic interpretation, Kaymaz et al. demonstrated that carotid tortuosity prolongs internal carotid artery access time, while Koge et al. reported an association between ICA tortuosity and less favorable thrombectomy performance, including reduced FPE and prolonged procedural time [13,14]. The results obtained in our study, particularly with respect to the procedure time and the first-pass success, support these findings. The anatomical rationale for combining these features is that they describe complementary aspects of the catheter access route. Bovine arch configuration describes supra-aortic branching anatomy, aortic arch type characterizes arch geometry, and ICA dolichoarteriopathy identifies kinking, tortuosity, or coiling along the carotid pathway. These characteristics may influence catheter navigation and stable positioning, providing a procedural rationale for their joint assessment [3,4]. In the exploratory component-specific analyses, Type III aortic arch morphology and ICA dolichoarteriopathy were independently associated with longer puncture-to-recanalization time, whereas bovine arch configuration and Type II aortic arch morphology were not. None of the individual components showed a statistically significant independent association with FPE or 90-day functional independence. These findings suggest that the procedural association of the composite B.A.D. score may be driven particularly by Type III arch anatomy and ICA dolichoarteriopathy; however, because these analyses were exploratory and some anatomical subgroups were small, they should be interpreted cautiously and require external confirmation.

The fact that FPE was lower shows that poor B.A.D. anatomy may not only prolong the procedure but may also decrease the technical efficiency of the thrombectomy process. Huo et al. [15] reported that, in anterior circulation large vessel occlusions, functional independence was higher and procedure-related complications were lower in the patients in whom the FPE was obtained. Procedural complication rates did not differ significantly between the B.A.D. groups (6.3% vs. 9.1%; *p* = 0.446). The recorded complications included vasospasm, vessel rupture, and dissection. However, the relatively small number of events limits the ability to detect differences in uncommon procedural complications, and the absence of a statistically significant difference should not be interpreted as evidence of equivalent procedural risk. In our study as well, poor B.A.D. anatomy was associated with lower first-pass success after adjustment for the covariates included in the model. Female sex was also associated with a higher likelihood of FPE in the multivariable model. This finding was not based on a prespecified hypothesis and should therefore be regarded as exploratory. The present study was not designed to investigate potential sex-related mechanisms underlying this association, and residual confounding cannot be excluded. Independent confirmation in dedicated studies is warranted. However, the fact that the final mTICI ≥ 2b, mTICI ≥ 2c, and mTICI 3 rates were not different between the B.A.D. groups shows that, despite the initial anatomical difficulty, the final recanalization success could be obtained at similar levels in the two groups.

Patients in the poor B.A.D. group were older than those in the good B.A.D. group (mean age, 71.79 versus 67.17 years; Table 2). Age was included as a continuous covariate in all multivariable models. The associations of poor B.A.D. status with longer puncture-to-recanalization time and lower FPE success persisted after adjustment for age and the other included baseline covariates. However, this adjustment accounts for age only within the specified model assumptions. Residual confounding from unmeasured age-related characteristics or an incompletely captured age–outcome relationship cannot be excluded; therefore, the findings should not be interpreted as fully separating anatomical complexity from age-related influences.

In our study, although the 90-day functional independence rate was lower in the poor B.A.D. group, the difference was not statistically significant (51.9% vs. 63.6%; *p* = 0.092), and no statistically significant independent association between B.A.D. status and 90-day functional independence was demonstrated in the multivariable analyses. This finding differs from the relationship between B.A.D. and clinical outcome reported by Snelling et al. [3]. This difference may be related to the fact that our study had a more homogeneous patient group, including only proximal M1 occlusions, and to the fact that current thrombectomy techniques and devices may have decreased the effect of anatomical access difficulty on the final recanalization. In addition, the fact that the B.A.D. score, although it evaluates the extracranial access anatomy, does not cover many variables that directly affect the clinical outcome, such as the baseline NIHSS, the infarct burden, the thrombus characteristics, and the recanalization quality, may also explain this situation.

In contrast, the procedure time and the recanalization quality were more clearly associated with the clinical outcome. In separate multivariable models, a longer puncture-to-recanalization time was found to be associated with a lower probability of 90-day functional independence, and mTICI ≥ 2c recanalization with a higher probability of functional independence. Lee et al. [16] reported in a large real-life cohort that a shorter endovascular procedure time was associated with a better functional outcome. Similarly, in the systematic review and meta-analysis published by Gao et al. in 2026 [17], it was shown that mTICI 2c–3 recanalization was associated with higher functional independence and lower mortality compared with mTICI 2b. Difficult vascular access itself may also be clinically relevant. Ribó et al. reported that difficult catheter access to the target carotid artery was associated with lower recanalization rates and worse 3-month functional outcomes, supporting a potential link between access complexity and long-term clinical outcome [18]. However, it should be taken into consideration that the long-term clinical outcomes in acute ischemic stroke cannot be explained only by the procedural success or by the vascular anatomical characteristics, and that post-procedural systemic and clinical factors may also affect the outcomes. Indeed, Kuki et al. reported that the post-procedural serum sodium levels were associated with 90-day mortality in patients with acute ischemic stroke who underwent mechanical thrombectomy [19]. When these findings are evaluated together, the B.A.D. score may serve as a marker of anatomical complexity encountered during the recanalization process and may have potential value for future procedural planning.

Our findings raise the hypothesis that the possible clinical effect of the B.A.D. anatomy may emerge through procedural steps such as a prolonged procedure time and a decreased FPE success. However, because a mediation analysis was not performed in our study, this possible mechanism has not been demonstrated directly. In addition, in the exploratory subgroup analysis of the patients in whom mTICI ≥ 2c recanalization was provided, no significant difference was detected between the B.A.D. groups with respect to the 90-day functional outcomes and mortality. Although this finding suggests that the clinical effect of the initial vascular anatomical difference may decrease when high-quality recanalization is obtained, it should be evaluated as hypothesis-generating in nature, since an interaction analysis was not performed.

The B.A.D. score may provide a structured approach to describing vascular access complexity on preprocedural CT angiography. Potential prospective applications include anticipating catheter-support requirements, informing device selection and preparation, and considering alternative access options earlier in procedural planning. Its possible contribution to triage could involve anticipating procedural support and resource requirements. These represent potential applications for future evaluation rather than benefits demonstrated in the present cohort.

Because the present cohort included only transfemoral procedures, our data cannot determine whether patients with unfavorable B.A.D. anatomy would benefit from an alternative access route. Nevertheless, preprocedural identification of complex arch and cervical anatomy may help identify patients in whom transradial or direct carotid access could be considered earlier when transfemoral access is expected to be challenging. This potential application remains hypothesis-generating and requires prospective comparison of access strategies before the B.A.D. score can be used to guide access selection. Independent clinical series have also supported transradial access as a feasible alternative to transfemoral thrombectomy in selected patients with anterior circulation stroke [20].

Direct carotid puncture has also been described as an alternative strategy in patients with prohibitive vascular access [21].

The external validity of the B.A.D. score remains uncertain, as vascular anatomy distributions, operator experience, device selection, and thrombectomy workflows may differ across institutions and patient populations. Therefore, independent validation in larger multicenter cohorts is an essential next step before the score can be considered for broader clinical implementation.

The present study did not compare B.A.D.-guided strategies or evaluate their impact on device selection, access optimization, or triage decisions. Therefore, the findings do not establish predictive performance or clinical benefit sufficient to support routine implementation as a decision-making tool. In particular, the score should not be interpreted as a stand-alone criterion for determining thrombectomy eligibility or selecting a specific device or access route. Independent prospective validation and evaluation of whether score-guided planning improves procedural or clinical outcomes are needed.

## 5. Limitations

This single-center retrospective study is subject to residual confounding despite multivariable adjustment. Although the additional models accounted for major procedural factors available in our dataset, including thrombectomy technique and the number of thrombectomy passes, other potentially relevant procedural, device-specific, and operator-related factors could not be fully accounted for. In particular, detailed information on specific device selection, rescue procedures, and operator preference was not consistently available and therefore could not be incorporated into the analyses. Although no balloon-guide catheters were used and all aspiration catheters were 6F in size, the lack of variation in these characteristics precluded evaluation of their potential influence on procedural outcomes. These factors may have influenced FPE and procedural duration independently of vascular anatomy. Consequently, although the association between B.A.D. and procedural outcomes persisted after adjustment for the available clinical and procedural covariates, residual confounding cannot be excluded, and the observed associations should not be interpreted as causal.

Although B.A.D. scoring was performed by an experienced interventional neurologist who was blinded to procedural and clinical outcomes, the assessment relied on a single reader, and inter-rater reproducibility was not evaluated. Blinding does not address potential observer-dependent variability in anatomical classification, particularly for aortic arch type and ICA dolichoarteriopathy. The reproducibility of B.A.D. assessment in this cohort therefore remains unestablished.

All procedures were performed by a single interventional neurologist. Therefore, between-operator variability could not be assessed, and the findings may reflect operator-specific practices. No balloon-guide catheters were used, and all aspiration catheters had an inner lumen diameter of 6F. Generalizability to centers using balloon-guide catheters, aspiration catheters with different inner lumen diameters, or alternative thrombectomy workflows requires further evaluation.

The restriction to proximal MCA M1 occlusions treated through transfemoral access, together with the exclusion of patients with intubation or general anesthesia, limits applicability to other patient groups. Excluding patients with missing required clinical, imaging, or outcome data may also have introduced selection bias. Furthermore, the B.A.D. score does not encompass all supra-aortic anatomical characteristics. Although the cohort comprised all consecutively eligible patients during the study period, the sample size may have limited statistical power to detect modest but clinically relevant differences in 90-day functional outcomes. Therefore, the non-significant difference in functional independence between the B.A.D. groups should not be interpreted as evidence of equivalence or absence of an association.

Furthermore, the cutoff used to dichotomize the B.A.D. score has not been formally validated as a prognostic threshold. Although sensitivity analyses using the original ordinal score supported the association with procedural duration, the component-specific analyses were exploratory and some anatomical subgroups were relatively small. These findings therefore require external validation in larger cohorts.

The present study was not designed to validate the standalone predictive performance of the B.A.D. score. Dedicated measures of discrimination and calibration, including ROC/AUC analysis, threshold-specific sensitivity and specificity, and predictive values, were therefore not assessed. Consequently, the observed associations should not be interpreted as validation of an individual-level clinical prediction tool.

## 6. Conclusions

In conclusion, poor B.A.D. anatomy was associated with greater procedural complexity during mechanical thrombectomy for proximal MCA M1 occlusion, including longer puncture-to-recanalization time and lower odds of FPE success after adjustment for available clinical and procedural factors. Final recanalization rates did not differ significantly between the B.A.D. groups, and no statistically significant independent association between B.A.D. status and 90-day functional independence was demonstrated. These findings suggest that the B.A.D. score may have potential value as a preprocedural marker of vascular access complexity; however, prospective external validation is required before its use for guiding access selection or procedural strategy can be recommended.

## Figures and Tables

**Table 1:** Distribution of Anatomical Components of the B.A.D. Score According to B.A.D. Group.

Variable		Total (*n* = 220)	Good B.A.D. (<2) (*n* = 143)	Poor B.A.D. (≥2) (*n* = 77)
Bovine arch, present		31 (14.1%)	0 (0.0%)	31 (40.3%)
Aortic arch type				
	Type I	29 (13.2%)	29 (20.3%)	0 (0.0%)
	Type II	141 (64.1%)	114 (79.7%)	27 (35.1%)
	Type III	50 (22.7%)	0 (0.0%)	50 (64.9%)
ICA dolichoarteriopathy score				
	0 (none or mild kinking)	188 (85.5%)	143 (100.0%)	45 (58.4%)
	1 (moderate or severe kinking or tortuosity or coiling)	32 (14.5%)	0 (0.0%)	32 (41.6%)

Footnotes: Data are presented as *n* (%). Percentages were calculated within each respective column. B.A.D. scores of 0–1 were classified as good, whereas scores ≥2 were classified as poor. Abbreviations: B.A.D., Bovine Aortic Arch–Dolichoarteriopathy; ICA, internal carotid artery.

**Table 2:** Baseline Demographic and Clinical Characteristics According to B.A.D. Group.

Variable	Total (*n* = 220)	Good B.A.D. (<2) (*n* = 143)	Poor B.A.D. (≥2) (*n* = 77)	*p* Value
Age (years)	68.79 ± 12.29	67.17 ± 12.90	71.79 ± 10.51	**0.007**
Female sex	121 (55.0%)	77 (53.8%)	44 (57.1%)	0.639
Current smoker	39 (17.7%)	26 (18.2%)	13 (16.9%)	0.810
Ex-smoker	14 (6.4%)	7 (4.9%)	7 (9.1%)	0.253 *
Chronic alcohol use	11 (5.0%)	6 (4.2%)	5 (6.5%)	0.522 *
Hypertension	145 (65.9%)	94 (65.7%)	51 (66.2%)	0.941
Diabetes mellitus	59 (26.8%)	38 (26.6%)	21 (27.3%)	0.911
Obesity	31 (14.1%)	18 (12.6%)	13 (16.9%)	0.382
Dyslipidemia	100 (45.5%)	68 (47.6%)	32 (41.6%)	0.394
Atrial fibrillation	127 (57.7%)	82 (57.3%)	45 (58.4%)	0.875
Coronary artery disease	69 (31.4%)	47 (32.9%)	22 (28.6%)	0.512
Previous CABG	13 (5.9%)	9 (6.3%)	4 (5.2%)	1.000 *
Peripheral artery disease	5 (2.3%)	3 (2.1%)	2 (2.6%)	1.000 *
Current malignancy	9 (4.1%)	5 (3.5%)	4 (5.2%)	0.723 *
Previous stroke/TIA	42 (19.1%)	32 (22.4%)	10 (13.0%)	0.091
Antiplatelet use	86 (39.1%)	56 (39.2%)	30 (39.0%)	0.977
OAC use	29 (13.2%)	17 (11.9%)	12 (15.6%)	0.440
Admission SBP, mmHg	152.12 ± 32.97	151.59 ± 32.18	153.09 ± 34.58	0.749
Admission DBP, mmHg	87.26 ± 18.06	87.29 ± 17.24	87.19 ± 19.61	0.969

Footnotes: Continuous variables are presented as mean ± standard deviation (SD), and categorical variables as *n* (%). Comparisons were performed using the independent-samples *t*-test for continuous variables and Pearson’s chi-square test or Fisher’s exact test, as appropriate. * Fisher’s exact test was used. Bold *p*-values indicate statistical significance (*p* < 0.05). Abbreviations: B.A.D., Bovine Aortic Arch Dolichoarteriopathy; CABG, coronary artery bypass grafting; TIA, transient ischemic attack; OAC, oral anticoagulant; SBP, systolic blood pressure; DBP, diastolic blood pressure; SD, standard deviation.

**Table 3:** Baseline Stroke, Imaging, and Treatment Characteristics According to B.A.D. Group.

Variable	Total (*n* = 220)	Good B.A.D. (<2) (*n* = 143)	Poor B.A.D. (≥2) (*n* = 77)	*p* Value
Admission NIHSS score	17 (14–19)	17 (14–18)	17 (14–19)	0.847
Admission ASPECTS	9 (8–10)	9 (8–10)	9 (8–10)	0.583
IV thrombolysis	26 (11.8%)	15 (10.5%)	11 (14.3%)	0.405
Right hemisphere	111 (50.5%)	72 (50.3%)	39 (50.6%)	0.966
Good collateral status	170 (77.27%)	111 (77.62%)	59 (76.62%)	0.927

Footnotes: Continuous variables are presented as median (interquartile range [IQR]), and categorical variables as *n* (%). Continuous variables were compared using the Mann–Whitney U test, and categorical variables using Pearson’s chi-square test. Good collateral status was defined as a mTAN collateral score of 2–3, whereas mTAN scores of 0–1 were classified as poor collateral status. Abbreviations: B.A.D., Bovine Aortic Arch Dolichoarteriopathy; NIHSS, National Institutes of Health Stroke Scale; ASPECTS, Alberta Stroke Program Early CT Score; IV, intravenous; mTAN, Modified Tan collateral score; IQR, interquartile range.

**Table 4:** Distribution of Stroke Etiologies According to B.A.D. Group.

Variable	Total (*n* = 220)	Good B.A.D. (<2) (*n* = 143)	Poor B.A.D. (≥2) (*n* = 77)	*p* Value
Large artery atherosclerosis	18 (8.2%)	11 (7.7%)	7 (9.1%)	0.849
Cardioembolism	142 (64.5%)	91 (63.6%)	51 (66.2%)	
Other determined etiology	13 (5.9%)	8 (5.6%)	5 (6.5%)	
Undetermined etiology	47 (21.4%)	33 (23.1%)	14 (18.2%)	

Footnotes: Data are presented as *n* (%). The overall distribution of stroke etiologies between B.A.D. groups was compared using Pearson’s chi-square test. Abbreviations: B.A.D., Bovine Aortic Arch Dolichoarteriopathy.

**Table 5:** Procedural Characteristics and Recanalization Outcomes According to B.A.D. Group.

Variable	Total (*n* = 220)	Good B.A.D. (<2) (*n* = 143)	Poor B.A.D. (≥2) (*n* = 77)	*p* Value
Symptom-to-recanalization time, min	223.5 (182.8–311.8)	221.0 (182.5–325.5)	228.0 (186.0–309.0)	0.631
Puncture-to-recanalization time, min	51.0 (36.0–71.0)	43.0 (32.0–65.0)	67.0 (50.5–88.0)	**<0.001**
Symptom-to-puncture time, min	165.0 (125.0–247.8)	177.0 (126.0–254.0)	160.0 (120.0–239.5)	0.313
First-pass effect	103 (46.8%)	74 (51.7%)	29 (37.7%)	**0.046**
≥3 passes	53 (24.1%)	33 (23.1%)	20 (26.0%)	0.632
Number of passes	2 (1–2)	1 (1–2)	2 (1–3)	0.075
mTICI ≥ 2b	204 (92.7%)	135 (94.4%)	69 (89.6%)	0.191
mTICI ≥ 2c	184 (83.6%)	123 (86.0%)	61 (79.2%)	0.194
mTICI 3	130 (59.1%)	90 (62.9%)	40 (51.9%)	0.114
Combined thrombectomy technique	133 (60.5%)	88 (61.5%)	45 (58.4%)	0.654

Footnotes: Continuous variables are presented as median (interquartile range [IQR]) and categorical variables as *n* (%). Continuous variables were compared using the Mann–Whitney U test, and categorical variables using Pearson’s chi-square test. Combined thrombectomy technique was defined as the use of contact aspiration together with a stent retriever rather than aspiration alone. FPE was defined as complete recanalization (mTICI 3) after a single thrombectomy pass without rescue treatment. Bold *p*-values indicate statistical significance (*p* < 0.05). Abbreviations: B.A.D., Bovine Aortic Arch Dolichoarteriopathy; IQR, interquartile range; mTICI, modified Thrombolysis in Cerebral Infarction.

**Table 6:** Ninety-Day Clinical Outcomes and Complications According to B.A.D. Group.

Variable	Total (*n* = 220)	Good B.A.D. (<2) (*n* = 143)	Poor B.A.D. (≥2) (*n* = 77)	*p* Value
90-day mRS score	2 (0–5)	2 (0–5)	2 (0–5)	0.316
90-day mRS 0–1	104 (47.3%)	71 (49.7%)	33 (42.9%)	0.336
90-day mRS 0–2	131 (59.5%)	91 (63.6%)	40 (51.9%)	0.092
90-day mortality (mRS 6)	45 (20.5%)	29 (20.3%)	16 (20.8%)	0.930
Symptomatic intracerebral hemorrhage	21 (9.5%)	15 (10.5%)	6 (7.8%)	0.516
Other procedural complications *	16 (7.3%)	9 (6.3%)	7 (9.1%)	0.446

Footnotes: Continuous variables are presented as median (interquartile range [IQR]) and categorical variables as *n* (%). Continuous variables were compared using the Mann–Whitney U test, and categorical variables using Pearson’s chi-square test. * Other procedural complications included vasospasm (*n* = 14), vessel rupture (*n* = 1), and dissection (*n* = 2). Because vasospasm and dissection occurred concurrently in one patient, the total number of patients with other procedural complications was 16 rather than 17. Abbreviations: B.A.D., Bovine Aortic Arch Dolichoarteriopathy; IQR, interquartile range; mRS, modified Rankin Scale.

**Table 7:** Hematologic and Biochemical Parameters According to B.A.D. Group.

Variable	Total (*n* = 220)	Good B.A.D. (<2) (*n* = 143)	Poor B.A.D. (≥2) (*n* = 77)	*p* Value
Hemoglobin, g/dL	12.31 ± 1.84	12.37 ± 1.93	12.21 ± 1.66	0.523
Platelet count, ×10^3^/µL	240 (193–291)	235 (190–284)	254 (204–302)	0.121
Leukocyte count, /µL	9320 (7530–11,590)	9500 (7720–11,700)	9080 (7240–11,290)	0.149
Neutrophil count, /µL	6730 (5120–8790)	6810 (5250–9010)	6580 (4880–8510)	0.197
Lymphocyte count, /µL	1650 (1190–2160)	1710 (1240–2230)	1530 (1100–2000)	0.107
Monocyte count, /µL	630 (490–810)	630 (490–810)	620 (490–810)	0.945
Glucose, mg/dL	126 (105–159)	126 (105–158)	126 (105–160)	0.920
Total cholesterol, mg/dL	172.21 ± 43.20	174.44 ± 43.29	168.12 ± 43.11	0.306
LDL-C, mg/dL	113.11 ± 34.38	114.80 ± 34.43	109.98 ± 34.32	0.325
HDL-C, mg/dL	43.54 ± 11.47	43.44 ± 12.01	43.72 ± 10.46	0.864
Triglycerides, mg/dL	77.50 (56.04–106.00)	75.50 (56.04–106.00)	81.00 (56.25–107.25)	0.672
BUN, mg/dL	18.00 (14.00–23.00)	18.00 (14.00–23.00)	18.00 (14.00–24.00)	0.723
Creatinine, mg/dL	0.90 (0.75–1.10)	0.90 (0.75–1.10)	0.90 (0.74–1.08)	0.531
eGFR, mL/min/1.73 m^2^	78.00 (62.00–92.00)	79.00 (63.00–93.00)	76.00 (60.00–91.00)	0.488
CRP, mg/L	5.40 (2.20–16.00)	5.00 (2.10–14.00)	6.30 (2.40–20.00)	0.217
INR	1.08 (1.01–1.17)	1.08 (1.01–1.17)	1.08 (1.01–1.17)	0.878

Footnotes: Continuous variables are presented as mean ± standard deviation (SD) or median (interquartile range [IQR]), as appropriate. Comparisons were performed using the independent-samples *t*-test or Mann–Whitney U test, as appropriate. Abbreviations: SD, standard deviation; IQR, interquartile range; B.A.D., Bovine Aortic Arch Dolichoarteriopathy; LDL-C, low-density lipoprotein cholesterol; HDL-C, high-density lipoprotein cholesterol; BUN, blood urea nitrogen; eGFR, estimated glomerular filtration rate; CRP, C-reactive protein; INR, international normalized ratio.

**Table 8:** Ninety-Day Clinical Outcomes According to B.A.D. Group Among Patients Achieving mTICI ≥ 2c Reperfusion.

90-Day Outcome	Total (*n* = 184)	Good B.A.D. (<2) (*n* = 123)	Poor B.A.D. (≥2) (*n* = 61)	*p* Value
90-day mRS	1 (0–3)	1 (0–3)	1 (0–3)	0.584
90-day mRS 0–1	103 (56.0%)	70 (56.9%)	33 (54.1%)	0.718
90-day mRS 0–2	127 (69.0%)	88 (71.5%)	39 (63.9%)	0.293
90-day mRS 6 (mortality)	31 (16.8%)	20 (16.3%)	11 (18.0%)	0.762

Footnotes: Data are presented as median (interquartile range [IQR]) or *n* (%), as appropriate. This subgroup analysis included only patients who achieved near-complete or complete reperfusion (mTICI ≥ 2c). The 90-day mRS distribution was compared between B.A.D. groups using the Mann–Whitney U test, whereas categorical outcomes were compared using Pearson’s chi-square test. Abbreviations: B.A.D., Bovine Aortic Arch Dolichoarteriopathy; mTICI, modified Thrombolysis in Cerebral Infarction; mRS, modified Rankin Scale; IQR, interquartile range.

**Table 9:** Univariate and Multivariable Logistic Regression Analyses of Factors Associated with First-Pass Effect.

Predictor	Unadjusted OR (95% CI)	*p*-Value	Adjusted OR (95% CI)	*p*-Value
Poor B.A.D. (≥2) [vs. Good B.A.D. (<2)]	0.563 (0.320–0.992)	**0.047**	0.439 (0.229–0.839)	**0.013**
Age, per 1-year increase	0.994 (0.973–1.016)	0.597	0.999 (0.974–1.024)	0.931
Female sex (vs. male)	2.171 (1.260–3.742)	**0.005**	2.158 (1.176–3.959)	**0.013**
Admission NIHSS, per 1-point increase	0.993 (0.927–1.063)	0.832	0.996 (0.920–1.078)	0.916
Admission ASPECTS, per 1-point increase	1.119 (0.865–1.448)	0.390	1.001 (0.739–1.354)	0.997
Good collateral status (mTAN 2–3 vs. 0–1)	1.305 (0.682–2.494)	0.421	1.037 (0.494–2.176)	0.924
Combined thrombectomy technique (vs. aspiration alone)	0.215 (0.120–0.385)	<0.001	0.199 (0.107–0.370)	<0.001

Footnotes: Binary logistic regression analyses were performed with first-pass effect success (yes = 1, no = 0) as the dependent variable. The multivariable model included B.A.D. group, age, sex, admission NIHSS score, admission ASPECTS, collateral status, and thrombectomy technique. Good B.A.D. (<2), male sex, poor collateral status (mTAN 0–1), and aspiration alone served as reference categories, as applicable. ORs for continuous variables represent the change in the odds of first-pass effect success per one-unit increase. The multivariable model was statistically significant (Omnibus χ^2^(7) = 42.043, *p* < 0.001); the Hosmer–Lemeshow goodness-of-fit test indicated adequate model fit (χ^2^(8) = 5.800, *p* = 0.670), and the Nagelkerke R^2^ was 0.234. Bold values indicate statistical significance (*p* < 0.05). Analyses were performed using complete cases for the variables included in each model; no imputation was performed. Abbreviations: B.A.D., Bovine Aortic Arch Dolichoarteriopathy; OR, odds ratio; CI, confidence interval; NIHSS, National Institutes of Health Stroke Scale; ASPECTS, Alberta Stroke Program Early CT Score; mTAN, Modified Tan collateral score.

**Table 10:** Univariate and Multivariable Logistic Regression Analyses of Factors Associated with 90-Day Functional Independence (mRS 0–2).

Predictor	Unadjusted OR (95% CI)	*p*-Value	Model 1 aOR (95% CI)	*p*-Value	Model 2 aOR (95% CI)	*p*-Value	Model 3 aOR (95% CI)	*p*-Value
Poor B.A.D. (≥2) vs. Good B.A.D. (<2)	0.618 (0.352–1.084)	0.093	0.578 (0.310–1.079)	0.085	0.615 (0.309–1.226)	0.167	0.896 (0.452–1.774)	0.752
Age, per 1-year increase	0.977 (0.955–1.000)	0.050	0.989 (0.964–1.015)	0.403	0.988 (0.960–1.017)	0.418	0.993 (0.967–1.021)	0.627
Female sex (vs. male)	1.252 (0.729–2.150)	0.416	1.296 (0.708–2.373)	0.400	1.081 (0.552–2.120)	0.820	1.134 (0.600–2.143)	0.699
Admission NIHSS, per 1-point increase	0.844 (0.776–0.918)	**<0.001**	0.849 (0.776–0.929)	**<0.001**	0.849 (0.769–0.937)	**0.001**	0.843 (0.768–0.926)	**<0.001**
Admission ASPECTS, per 1-point increase	1.326 (1.020–1.724)	**0.035**	1.250 (0.928–1.683)	0.142	1.176 (0.846–1.634)	0.335	1.233 (0.907–1.676)	0.181
IV thrombolysis (yes vs. no)	2.492 (0.959–6.481)	0.061	1.880 (0.667–5.297)	0.232	3.001 (0.883–10.195)	0.078	1.559 (0.546–4.446)	0.407
Good collateral status (mTAN 2–3 vs. 0–1)	2.567 (1.334–4.938)	**0.005**	2.034 (0.992–4.170)	0.053	2.261 (1.025–4.984)	**0.043**	1.966 (0.919–4.205)	0.082
mTICI ≥ 2c reperfusion (yes vs. no)	17.825 (6.021–52.771)	**<0.001**	—	—	21.562 (6.383–72.834)	**<0.001**	—	—
Symptom-to-recanalization time, per 1 min increase	0.999 (0.998–1.001)	0.276	—	—	—	—	—	—
Symptom-to-puncture time, per 1 min increase	1.000 (0.998–1.001)	0.723	—	—	—	—	—	—
Puncture-to-recanalization time, per 1 min increase	0.976 (0.966–0.987)	**<0.001**	—	—	—	—	0.977 (0.966–0.989)	**<0.001**

Footnotes: Binary logistic regression analyses were performed with 90-day functional independence, defined as mRS 0–2 (yes = 1, no = 0), as the dependent variable. Model 1 was adjusted for B.A.D. group, age, sex, admission NIHSS score, admission ASPECTS, IV thrombolysis, and collateral status. Model 2 included the same covariates as Model 1 with additional adjustment for mTICI ≥ 2c reperfusion. Model 3 included the same covariates as Model 1 with additional adjustment for puncture-to-recanalization time. Good B.A.D. (<2), male sex, no IV thrombolysis, poor collateral status (mTAN 0–1), and mTICI <2c served as the reference categories, as applicable. ORs for continuous variables represent the change in the odds of 90-day functional independence per one-unit increase. Bold values indicate statistical significance (*p* < 0.05). Dashes indicate variables not included in the respective multivariable model. Model performance: Model 1: Omnibus χ^2^(7) = 33.911, *p* < 0.001; Hosmer–Lemeshow χ^2^(8) = 10.594, *p* = 0.226; Nagelkerke R^2^ = 0.195. Model 2: Omnibus χ^2^(8) = 74.341, *p* < 0.001; Hosmer–Lemeshow χ^2^(8) = 9.125, *p* = 0.332; Nagelkerke R^2^ = 0.390. Model 3: Omnibus χ^2^(8) = 50.083, *p* < 0.001; Hosmer–Lemeshow χ^2^(8) = 7.958, *p* = 0.438. Analyses were performed using complete cases for the variables included in each model; no imputation was performed. Abbreviations: B.A.D., Bovine Aortic Arch Dolichoarteriopathy; OR, odds ratio; aOR, adjusted odds ratio; CI, confidence interval; mRS, modified Rankin Scale; NIHSS, National Institutes of Health Stroke Scale; ASPECTS, Alberta Stroke Program Early CT Score; IV, intravenous; mTAN, Modified Tan collateral score; mTICI, modified Thrombolysis in Cerebral Infarction.

**Table 11:** Predictors of puncture-to-recanalization time.

Variable	Univariate B (95% CI)	*p* Value	Model 1 B (95% CI)	*p* Value	Model 2 B (95% CI)	*p* Value
Poor B.A.D. (≥2 vs. <2)	0.357 (0.223–0.490)	**<0.001**	0.338 (0.203–0.473)	**<0.001**	0.294 (0.188–0.400)	**<0.001**
Age, years	0.007 (0.001–0.013)	**0.017**	0.004 (−0.001–0.010)	0.125	0.005 (0.001–0.009)	**0.019**
Female sex (vs. male) †	−0.095 (−0.229–0.039)	0.166	−0.124 (−0.253–0.006)	0.061	−0.011 (−0.113–0.091)	0.833
Baseline NIHSS	0.007 (−0.010–0.025)	0.408	0.004 (−0.013–0.021)	0.609	0.002 (−0.012–0.015)	0.812
Baseline ASPECTS	−0.052 (−0.116–0.012)	0.114	−0.031 (−0.095–0.033)	0.341	−0.022 (−0.072–0.028)	0.390
IV thrombolysis (yes vs. no)	−0.129 (−0.336–0.078)	0.219	−0.113 (−0.313–0.087)	0.267	−0.081 (−0.237–0.074)	0.303
Good collateral status (mTAN 2–3 vs. 0–1)	−0.059 (−0.222–0.104)	0.475	−0.010 (−0.170–0.149)	0.901	0.026 (−0.098–0.150)	0.678
Number of passes, per 1-pass increase	0.229 (0.188–0.270)	**<0.001**	—	—	0.208 (0.168–0.248)	**<0.001**
Combined thrombectomy technique (vs. aspiration alone)	0.327 (0.197–0.457)	**<0.001**	—	—	0.137 (0.030–0.245)	**0.013**

Footnotes: Values are presented as unstandardized regression coefficients (B) with 95% confidence intervals (CI). The dependent variable was the natural logarithm-transformed puncture-to-recanalization time (LN_PONK_REKAN). Positive B coefficients indicate longer puncture-to-recanalization time, whereas negative coefficients indicate shorter time. Univariate linear regression analyses were performed separately for each predictor. Model 1 included B.A.D. group, age, sex, baseline NIHSS, baseline ASPECTS, IV thrombolysis, and collateral status. Model 2 additionally included the number of thrombectomy passes and thrombectomy technique (combined technique vs. aspiration alone) to account for major procedural factors. All variables were entered simultaneously using the Enter method. Model 1 was statistically significant [F(7,210) = 5.344, *p* < 0.001; R^2^ = 0.151; adjusted R^2^ = 0.123]. Model 2 was also statistically significant [F(9,208) = 22.667, *p* < 0.001; R^2^ = 0.495; adjusted R^2^ = 0.473]. No evidence of problematic multicollinearity was observed in Model 2 (VIF range, 1.058–1.171). Good B.A.D. (<2), male sex, no IV thrombolysis, poor collateral status (mTAN 0–1), and aspiration alone served as reference categories, as applicable. † Sex was coded as 0 = female and 1 = male in the original dataset; coefficients and confidence limits were sign-reversed for presentation as female vs. male, while *p* values were unchanged. Bold values indicate statistical significance (*p* < 0.05). Dashes indicate variables not included in Model 1. Analyses were performed using complete cases for the variables included in each model; no imputation was performed. Abbreviations: B.A.D., bovine aortic arch dolichoarteriopathy; NIHSS, National Institutes of Health Stroke Scale; ASPECTS, Alberta Stroke Program Early CT Score; IV, intravenous; mTAN, Modified Tan collateral score; CI, confidence interval.

## Data Availability

The data presented in this study are available upon reasonable request from the corresponding author. The data are not publicly available due to privacy and ethical restrictions.

## Supplementary Materials

The following supporting information can be downloaded at: https://www.mdpi.com/article/10.3390/medicina62101933/s1, Supplementary Table S1: Sensitivity analyses treating the B.A.D. score as an ordinal variable; Supplementary Table S2: Component-Specific Analyses of the B.A.D. Score for Procedural and Clinical Outcomes.

## Abbreviations

The following abbreviations are used in this manuscript:

AIS	Acute Ischemic Stroke
aOR	Adjusted Odds Ratio
ASPECTS	Alberta Stroke Program Early CT Score
B.A.D.	Bovine Aortic Arch–Dolichoarteriopathy
BUN	Blood Urea Nitrogen
CABG	Coronary Artery Bypass Grafting
CI	Confidence Interval
CRP	C-Reactive Protein
CT	Computed Tomography
DBP	Diastolic Blood Pressure
eGFR	Estimated Glomerular Filtration Rate
FPE	First-Pass Effect
HDL-C	High-Density Lipoprotein Cholesterol
HIMS	Hospital Information Management System
ICA	Internal Carotid Artery
INR	International Normalized Ratio
IQR	Interquartile Range
IV	Intravenous
LDL-C	Low-Density Lipoprotein Cholesterol
LVO	Large Vessel Occlusion
MCA	Middle Cerebral Artery
mRS	Modified Rankin Scale
MT	Mechanical Thrombectomy
mTAN	Modified TAN Collateral Score
mTICI	Modified Thrombolysis in Cerebral Infarction
NIHSS	National Institutes of Health Stroke Scale
OAC	Oral Anticoagulant
OR	Odds Ratio
PACS	Picture Archiving and Communication System
SBP	Systolic Blood Pressure
SD	Standard Deviation
TIA	Transient Ischemic Attack
TOAST	Trial of Org 10172 in Acute Stroke Treatment
