# Supplementary material for: Vascular Access Complexity and Thrombectomy Efficiency in Proximal MCA M1 Occlusion: Evaluation of the Bovine Aortic Arch–Dolichoarteriopathy Score
Source: Medicina (Kaunas). 2026 Oct 7;62(10):1933. doi: 10.3390/medicina62101933 (PMC13707307; doi:10.3390/medicina62101933)
Supplement: Supplementary file 1 [file medicina-62-01933-s001.zip › medicina-4605427-supplementary.pdf]

**Supplementary Table S1.** Sensitivity analyses treating the B.A.D. score as an ordinal variable

| Outcome | Model | Effect estimate per 1-point increase in B.A.D. score | 95% CI | p value |
|---|---|---|---|---|
| First-pass recanalization | Adjusted model | aOR 0.721 | 0.514–1.010 | 0.057 |
| Puncture-to-recanalization time† | Model 1 | B 0.181 | 0.110–0.251 | **<0.001** |
| | Model 2 | B 0.164 | 0.110–0.218 | **<0.001** |
| 90-day functional independence (mRS 0–2) | Model 1 | aOR 0.767 | 0.549–1.070 | 0.118 |
| | Model 2 | aOR 0.814 | 0.560–1.182 | 0.279 |
| | Model 3 | aOR 0.984 | 0.682–1.420 | 0.932 |

***Footnotes:*** *Sensitivity analyses were performed by treating the B.A.D. score as an ordinal variable ranging from 0 to 4, with effect estimates reported per 1-point increase in the score. For first-pass recanalization, multivariable logistic regression analysis was adjusted for age, sex, baseline NIHSS, baseline ASPECTS, collateral status, and thrombectomy technique (combined technique vs aspiration alone). For puncture-to-recanalization time, multivariable linear regression analyses were performed using the natural logarithm-transformed puncture-to-recanalization time as the dependent variable because of its right-skewed distribution. Model 1 was adjusted for age, sex, baseline NIHSS, baseline ASPECTS, IV thrombolysis, and collateral status. Model 2 additionally included the number of thrombectomy passes and thrombectomy technique (combined technique vs aspiration alone). For 90-day functional independence, multivariable logistic regression analyses were performed with mRS 0–2 as the favorable outcome. Model 1 was adjusted for age, sex, baseline NIHSS, baseline ASPECTS, IV thrombolysis, and collateral status; Model 2 additionally included final mTICI ≥2c; and Model 3 additionally included puncture-to-recanalization time instead of final mTICI ≥2c. In the logistic regression analyses, aORs <1 indicate lower odds of the respective favorable outcome per 1-point increase in B.A.D. score. For the linear regression analyses, positive B coefficients indicate longer puncture-to-recanalization time on the logarithmic scale. Bold values indicate statistical significance ($p < 0.05$).*

*† Puncture-to-recanalization time was natural logarithm-transformed before linear regression analysis.*

***Abbreviations:*** *B, unstandardized regression coefficient; B.A.D., Bovine Aortic Arch Dolichoarteriopathy; CI, confidence interval; mRS, modified Rankin Scale; aOR, adjusted odds ratio.*

**Supplementary Table S2.** Component-Specific Analyses of the B.A.D. Score for Procedural and Clinical Outcomes

| B.A.D. component | Puncture-to-recanalization time, Adjusted B (95% CI) | p value | First-pass recanalization, Adjusted OR (95% CI) | p value | 90-day functional independence, Adjusted OR (95% CI) | p value |
|---|---|---|---|---|---|---|
| Bovine arch (yes vs no) | 0.011 (−0.143–0.166) | 0.884 | 1.688 (0.640–4.451) | 0.290 | 1.029 (0.400–2.652) | 0.952 |
| Aortic arch Type II (vs Type I) | 0.063 (−0.097–0.223) | 0.438 | 1.421 (0.521–3.875) | 0.492 | 0.897 (0.315–2.554) | 0.839 |
| Aortic arch Type III (vs Type I) | 0.409 (0.207–0.612) | **<0.001** | 0.453 (0.125–1.637) | 0.227 | 0.628 (0.174–2.269) | 0.478 |
| ICA dolichoarteriopathy (yes vs no) | 0.199 (0.047–0.351) | **0.010** | 0.419 (0.162–1.083) | 0.072 | 0.558 (0.227–1.374) | 0.204 |

***Footnotes:*** *Component-specific multivariable regression analyses were performed to explore the individual associations of the anatomical components of the B.A.D. score with procedural and clinical outcomes. Bovine arch, aortic arch type, and ICA dolichoarteriopathy were entered simultaneously into each model. Aortic arch Type I served as the reference category for aortic arch type.*

*For puncture-to-recanalization time, multivariable linear regression was performed using natural logarithm-transformed puncture-to-recanalization time as the dependent variable. The model was additionally adjusted for age, sex, baseline NIHSS, baseline ASPECTS, IV thrombolysis, collateral status, number of thrombectomy passes, and thrombectomy technique. Values are presented as unstandardized regression coefficients (B) with 95% CIs; positive B coefficients indicate longer puncture-to-recanalization time.*

*For first-pass recanalization, multivariable binary logistic regression was additionally adjusted for age, sex, baseline NIHSS, baseline ASPECTS, collateral status, and thrombectomy technique. For 90-day functional independence (mRS 0–2), multivariable binary logistic regression was additionally adjusted for age, sex, baseline NIHSS, baseline ASPECTS, IV thrombolysis, and collateral status. Adjusted ORs >1 indicate higher odds and adjusted ORs <1 indicate lower odds of the respective favorable outcome. No, male sex, poor collateral status (TAN 0–1), no IV thrombolysis, and aspiration alone served as reference categories, as applicable. Bold values indicate statistical significance (p < 0.05).*

***Abbreviations:*** *B, unstandardized regression coefficient; B.A.D., Bovine Aortic Arch Dolichoarteriopathy; CI, confidence interval; ICA, internal carotid artery; OR, odds ratio.*
